# The Invisible Epidemic: A Spotlight on the Opioid Crisis Among Older Adults

**DOI:** 10.1093/geroni/igaa057.678

**Published:** 2020-12-16

**Authors:** Jessica Hsieh, Lynn McDonald, Adriana Shnall, Raza Mirza, Samir Sinha, Andrea Austen, Laura Tamblyn Watts, Christopher Klinger

**Affiliations:** 1 University of Toronto, Toronto, Ontario, Canada; 2 Baycrest Health Sciences, North York, Canada; 3 National Initiative for the Care of the Elderly, Toronto, Ontario, Canada; 4 Sinai Health, Toronto, Canada; 5 City of Toronto, Toronto, Ontario, Canada; 6 CanAge, Toronto, Canada

## Abstract

Canada is facing an opioid crisis, with more than 3,900 related deaths occurring in 2017. Almost 30% of those deaths were among seniors; older adults also have the highest rates of opioid hospitalization and poisoning. Despite these statistics, little is known about the specifics. A comprehensive scoping review following Arksey and O’Malley’s (2005) framework was conducted to establish the magnitude of the problem, describe treatment approaches alongside prevention, and identify implications for practice, policy and research. Eight major electronic databases in medicine and the social sciences were searched alongside the grey literature, and a stakeholder consultation convened to validate results. A total of 6,814 articles, reports and thesis were identified. Forty-five sources met inclusion criteria, the majority stemming from the United States. Most were literature reviews, cohort and cross-sectional studies, with almost half also taking a gendered approach. Four predominant themes emerged from the thematic content analysis: 1) Medical Applications of Opioids; 2) Problematic Opioid Use; 3) Treatment and Prevention Strategies; and 4) Recommendations. Data highlighted ‘the invisible epidemic’, with treatment strategies to be specifically tailored to this population in light of metabolic differences and drug interactions as part of aging. Seniors are part of the current epidemic, with tailored approaches needed to ensure adequate, evidence-based counteraction – including seniors’ voices alongside further research in this regard. Education and training for prescribers needs to be enhanced alongside cross-jurisdictional drug monitoring programs to avoid drug interactions and misuse/fraud.

